# Activation of odorant receptor in colorectal cancer cells leads to inhibition of cell proliferation and apoptosis

**DOI:** 10.1371/journal.pone.0172491

**Published:** 2017-03-08

**Authors:** Lea Weber, Klaudia Al-Refae, Juliane Ebbert, Peter Jägers, Janine Altmüller, Christian Becker, Stephan Hahn, Günter Gisselmann, Hanns Hatt

**Affiliations:** 1 Department of Cell Physiology, Ruhr-University Bochum, Bochum, Germany; 2 Institute of Cell Biology (Cancer Research), University of Duisburg-Essen, University Hospital, Essen, Germany; 3 Cologne Center for Genomics, University of Cologne, Cologne, Germany; 4 Department of Molecular Gastrointestinal Oncology, Ruhr-University Bochum, Bochum, Germany; Duke University, UNITED STATES

## Abstract

The analysis and functional characterization of ectopically expressed human olfactory receptors (ORs) is becoming increasingly important, as many ORs have been identified in several healthy and cancerous tissues. OR activation has been demonstrated to have influence on cancer cell growth and progression. Here, ORs were identified using RNA-Seq analyses and RT-PCR. We demonstrated the OR protein localization in HCT116 cells using immunocytochemistry (IHC). In order to analyze the physiological role of OR51B4, we deorphanized the receptor by the use of CRE-Luciferase assays, conducted calcium imaging experiments as well as scratch- and proliferation assays. Furthermore, western blot analyses revealed the involvement of different protein kinases in the ligand-dependent signaling pathway. Receptor knockdown via shRNA was used to analyze the involvement of OR51B4.

We identified OR51B4, which is highly expressed in the colon cancer cell line HCT116 and in native human colon cancer tissues. We deorphanized the receptor and identified Troenan as an effective ligand. Troenan stimulation of HCT116 cells has anti-proliferative, anti-migratory and pro-apoptotic effects, mediated by changes in the intracellular calcium level upon PLC activation. These effects cause changes in the phosphorylation levels of p38, mTor and Akt kinases. Knockdown of the receptor via shRNA confirmed the involvement of OR51B4.

This study emphasizes the importance of ectopically expressed ORs in the therapy for several diseases. The findings provide the basis for alternative treatments of colorectal cancer.

## Introduction

Colorectal cancer (CRC) is one of the most frequent causes of cancer-related mortality in the world. Primary surgery can achieve a cure rate of 50%. In the United States, 93,000 new cases of colon cancer occur annually [1]. It is known that G-Protein coupled receptors (GPCRs) influence many aspects of tumorigenesis, including invasion, proliferation, motility and several cancer-associated signaling pathways [2]. GPCRs are key regulators of several physiological processes and are suitable targets for the treatment of cancer, as well as other diseases. Olfactory receptors (ORs) belong to the largest gene family in the human genome, the GPCRs, and were identified and first described by Linda Buck and Richard Axel in 1991 [3]. More than 350 putative functional OR genes are involved in the detection and discrimination of a multitude of odorants [4–8]. Buck and Axel postulated exclusive expression of ORs in the olfactory epithelium, which was refuted by the finding of OR transcripts in several other human tissues [9]. A study by Flegel et al. confirmed and extended this analysis by using Next Generation Sequencing in combination with RT-PCR, so that the general expression of olfactory receptors in several human tissues can be considered proven [10]. The lack of ligands for the most ectopically expressed ORs is the major bottleneck in the investigation of the ORs function outside of the olfactory epithelium. Nevertheless, the function of a few deorphanized receptors could be clarified, e.g. the involvement in chemotaxis of sperms [11] and the serotonin release in enterochromaffin cells of the gut [12]. A recent publication identified an OR in keratinocytes as a mediator of the ligand-induced wound healing processes [13]. ORs are not only detected in healthy tissues but also in tumor tissues, where they can affect cancer cell proliferation [14], metastasis and cell invasiveness [15,16]. Some ORs show tumor-specific regulation, as shown for PSGR (OR51E2), which is highly expressed in prostate cancer cells but weakly expressed in normal prostate cells [17]. The activation of OR51E2 leads to inhibition of cancer cell proliferation [18] making it a novel tumor target for therapy. The paralog of PSGR, OR51E1, could be identified as a potential novel tumor marker for small intestine neuroendocrine carcinomas [19]. It was postulated as a novel target for diagnosis in somatostatin receptor-negative lung carcinoids [20]. ORs were also found in olfactory neuroblastoma [21].

Here, we demonstrate the expression of OR51B4 in colon cancer cells HCT116 and show that stimulating it with its ligand Troenan inhibits cell proliferation and induces apoptosis in colon cancer cells HCT116 via a calcium induced activation of Phospholipase C (PLC), which leads to the phosphorylation of p38 and a reduced phosphorylation of Akt. The physiological effects of Troenan stimulation were investigated by using different proliferation, scratch and apoptosis assays. This observation provides novel evidences supporting the functional impact of specifically expressed ORs in cancer pathogenesis in general and furthermore might provide innovative medical opportunities as OR51B4 might serve as a new tumor target for the treatment of colorectal cancer.

## Materials and methods

### Chemicals

All odorants, including Troenan, were purchased from Sigma Aldrich (Munich, Germany) and Henkel (Düsseldorf, Germany). The inhibitor L-cis-diltiazem was purchased from Abcam (Cambridge, MA, USA), SQ22.536, Thapsigargin and U-73122 were purchased from Sigma Aldrich, and the TRP-channel inhibitor Ruthenium red was purchased from Abcam. BTP-2, Mibefradil and Gallein were purchased from Tocris (Bristol, UK). Ringer’s solution used in the calcium imaging experiments contained 140 mM NaCl, 5 mM KCl, 2 mM CaCl_2_, 1 mM MgCl_2_ and 10 mM HEPES, pH 7.4. Forskolin used in the CRE-Luciferase assay was obtained from Sigma Aldrich. Like all tested odorants and inhibitors, it was prediluted in DMSO prior to usage.

### Tissue preparation and reverse transcriptase PCR (RT-PCR)

Human colon cancer tissues were obtained from Dr. Ubrig, Augusta-Krankenanstalt Bochum, and Prof. Hahn, Department of Molecular Gastrointestinal Oncology. The study was carried out in accordance with the Declaration of Helsinki. A Written informed consent was signed by each patient, who participated. All experiments were done in agreement with the ethics commission (Ethics committee of the Faculty of Medicine, Ruhr-University Bochum, ethic no.: 3841–10). Tumor tissues from primary colon adenocarcinomas were preserved in RNAlater. The tissue disruption was carried out using a Homogenizer (Precellys24) and 1.4 ceramics beads (Precellys®). The RNA was isolated from the tumor samples with the RNeasy Mini Kit (Qiagen, Hilden, Germany) according to the manufacturer’s protocol, including G-Eliminator columns and an additional on-column DNaseІ digestion. The expression of the detected ORs was validated using RT-PCR. The primers detect about 300 bp of the OR ORF. PCR was performed using the GoTaq qPCR Master Mix (Promega, Madison, WI, USA) according to the manufacturer’s protocol. For details see S1 Text.

### Next generation sequencing

We used the TruSeq^TM^ RNA Sample Prep Kit v2 protocol for standard mRNAseq (Illumina, San Diego, USA). RNA-Seq was performed on the HiSeq 2000 (2x102 bp reads) sequencing platform. Raw data were generated for HCT116 and SW982 cells and analyzed in fastq format. Reads were aligned to version hg19 of the human genome and transcriptome by using TopHat (v1.2.0) [22]. The alignment was arranged by the short-read mapping program Bowtie, included in TopHat [23]. Output files were in BAM format and were sorted and indexed by the program SAMtools [24]. To calculate the abundance of the aligned mRNA-Seq reads, the program Cufflinks was used [25]. It makes use of the RefSeq hg19 reference transcriptome in Gene Transfer Format (GTF) obtained from the UCSC Genome Bioinformatics database (University of California Santa Cruz), which was edited by the insertion of human olfactory pseudogenes [10]. Quantification of the transcript abundance of each gene was performed by Cufflinks. The transcript abundance is indicated in the unit FPKM (Fragments per Kilobase of exon per Million fragments mapped) [25]. The Integrative Genomic Viewer (IGV) was used to visualize the data sets and to analyze the read distribution (www.broasinstitute.org/igv).

### Published raw RNA-Seq data

The data sets used for RNA-Seq analysis were taken from NCBI. The data sets used for the comparison of HCT116 cell lines were taken from NCBI. Data sets for HCT116 cells were taken from following samples: GSM855450, GSM763960, GSM699333, GSE33480 and GSM898220. For comparison to our self-generated data, we reanalyzed the published raw data in the same manner as the RNA-Seq data for our own HCT116 sample. Data sets for other colon cancer cell lines were taken from the following studies: PRJNA183192, SAMEA2042211, SAMEA2042211, SAMEA2042213, SAMEA2042209; colon tumors and metastases: GSE46622; SH-EP and SK-N-SH cells: PRJNA205232, GSM981250; neuroblastoma tumors: SAMN00759066; breast tumors and cell lines: PRJNA80167, GSM1261022; bladder cancer: PRJNA185252, SAMN00627619; fibrosarcoma cell line: GSM985104; lung cancer and airway epithelium: GSE34914, SAMN00194131; melanoma cell lines: PRJNA39289; glioblastoma cell lines: GSE29738; lymphoblastoid cells: GSM002005.

### Expression plasmids

Vector construction and PCR amplification of OR51B4’s coding region:

For the transfection of HANA3A cells, an expression plasmid containing OR51B4 was constructed. Therefore, pCI mammalian expression vector was used (Promega). For PCR amplification of OR51B4 following Primers were used:

OR51B4-fwd: CGAGAATTCAGCATGTGGTATAACAACAGTGC

OR51B4-rev: CGAGCGGCCGCGCTTCAAGCCCTACTCTGCCC

Additionally, a PCR fragment consisting of the first 60 nucleotides of the coding region of the bovine rhodopsin (rhodopsin tag) was constructed and introduced between the BamHI and EcoRI sites. Standard PCR methods were used, the amplification protocol was: 1 x 1 min at 98°C, 40 x (10 s at 98°C, 30 s at 60°C, 30 s at 72°C) and 5 min at 72°C.

### Cell culture

HCT116, a well described [26] colon cancer cell line, was established from a human colorectal cancer and serves as a model cell line for the investigation of colorectal cancer. HCT116 cells and the reference cell lines Caco2, HT115 and SW620 were cultivated in Dulbecco’s Modified Eagle’s medium (DMEM) supplemented with 10% fetal bovine serum (FBS), glutamine (5%) and 100 units/ml penicillin/streptomycin (Gibco®, Life Technologies, Carlsbad, Ca, USA). The cells were cultured in a 6% CO_2_ humidified atmosphere at 37°C. Cells were passaged every 3–4 days at a confluence of 90% using TrypLE^TM^ (Life Technologies, Carlsbad, Ca, USA). Sarcoma cell line SW980 was provided by Prof. Steinsträsser, University Hospital Bergmannsheil, Bochum. The cells were cultured in DMEM containing 10% FBS and 100 units/ml Pen/Strep at 37°C and 6% CO_2_. Hana3A cells were used for the recombinant expression of ORs and were kindly provided by H. Matsunami (Duke University Medical Center, Durham, NC). Hana3A cells were cultured in DMEM containing 10% fetal bovine serum (FBS) and 100 units/ml penicillin/streptomycin (Gibco®, Life Technologies, Carlsbad, Ca, USA). They stably express RTP2, RTP1L, REEP1 and Gα_olf_, proteins that support the robust heterologous expression of different ORs [27].

### Immunocytochemistry

The transfection efficiency and the expression rate of the hOR51B4 protein in transfected Hana3A cells were measured by immunocytochemistry. The transfected cells were grown on coverslips in 24-well cell culture plates and fixed for 20 min in 4% paraformaldehyde at 4°C. After that, cells were washed and permeabilized in PBS^-/-^ + 0.1% Triton X-100. Blocking was conducted in PBST + 1% fish gelatin for 1 h at RT. The monoclonal anti-rhodopsin antibody 4D2 (anti-rho; Abcam; dilution 1:250) was incubated for 2 h at RT. Three washing steps were performed with washing solution, and the secondary antibody coupled to Alexa Fluor 488 (Invitrogen) was applied for 45 min at RT. Cells were washed three times and covered with Prolong® Gold Antifade reagent (Life Technologies). Images were taken with a Zeiss LSM 510 Meta confocal microscope. The localization of OR51B4 in HCT116 cells was visualized using a purified goat IgG polyclonal antibody against OR51B4 (Santa cruz, dilution: 1:100 in blocking solution) and mouse monoclonal anti-goat antibody (Abcam, dilution 1:1000 in blocking solution). For nuclear staining of the cells, 4´6-diamidino-2-phenylindole (DAPI; dilution 1:200) was applied.

### Deorphanization and pmirGLO Dual-Luciferase assay

For the recombinant expression of OR51B4, the coding sequence was amplified via PCR from human DNA and cloned into a pCI-vector downstream of the coding sequence for the 20 N-terminal amino acids of the human rhodopsin-Tag. We adapted the Dual-Luciferase assay optimized for odorant receptor screening by Zhuang and Matsunami [28]. In short: Hana3A cells were seeded (approximately 20,000 cells/well) and 24 hours later cells were transfected with FuGene (Promega) according to the manufacturer’s protocol using 18 μl FuGene, 5 μg hOR51B4, 1 μg RTP1s, 0.5 μg G_olf_, 2 μg pGL4-luciferase and 1 μg pRL-TK-Renilla plasmid for one 96-well plate. 24 hours after transfection, cells were stimulated with agonists diluted in CD293 with 2 mM L-glutamine for 4 hours at 37°C and 6% CO_2_. The Dual-Glo Luciferase Assay System (Promega) was used according to the manufacturer’s protocol. Cells transfected with only the pCI vector served as negative controls and excluded non-specific responses to the tested substances. For an effective screening procedure, we used Henkel 100 which contains one hundred odorous chemicals, including odorants from several classes and groups: aliphatics, alcohols, aromatics, amines, alkanes, aldehydes, esters, ethers, ketones, heterocyclics and others. The broad range of different substances enables the activation of as many ORs as possible in one single screening step. For more details and exact list of odorants see [29] and S2 Text.

### Calcium imaging

HCT116 cells grown in 35-mm dishes were incubated at 37°C for 30 minutes with Ringer’s solution (10 mM HEPES, 140 mM NaCl, 5 mM KCl, 2 mM CaCl_2_, 1 mM MgCl, pH 7.4) and 3 μM FURA-2-AM (fura-2-acetoxymethyl ester, Molecular Probes, Eugene, Oregon, USA). After the incubation, the growth medium was replaced with extracellular solution, and fluorimetric imaging was performed using a standard protocol [11]. HCT116 cells were exposed to 100–300 μM Troenan (2–3 times, 30–60 s, depending on the experimental approach). Troenan and other substances were prediluted in DMSO and dissolved in extracellular solution to their final concentrations. The final concentration of the DMSO solvent was 0.1% maximum. For experiments concerning the signaling pathway, inhibitors were co-applied (SQ22.536 (50 μM), L-cis-diltiazem (150 μM), Mibefradil (10 μM), H89 (10 μM), Ruthenium red (5 μM), BTP-2 (25 μM), Thapsigargin (1 μM), Gallein (10 μM)) or preincubated (U-73122 (10 μM)).

### Proliferation studies

Real Time Cell Analysis (RTCA) was performed using 16-well E-plates on a Dual Plate xCELLigence instrument (Roche Applied Science, Indianapolis IN). This system is capable of measuring a dimensionless parameter called cell index (CI), which evaluates the ionic environment at an electrode/solution interface and integrates information on cell number. 5,000 cells were seeded onto the E-plates in 200 μl of medium. After seeding, cells were allowed to settle for 30 min at RT before being inserted into the xCELLigence instrument in a humidified incubator at 37°C with 10% CO_2_. Medium supplemented with Troenan (50/100/150 μM) or DMSO was added to the cells after 24 hours. Continuous impedance measurements were then recorded every 15 min up to 200 hours. All assays were performed in duplicate.

For scratch assays, cells were seeded and grown in monolayers. After 24 hours, an artificial wound, a so-called “gap”, was created on the confluent cell monolayer by using a 20 μl pipette tip. The residual cells were washed with PBS^-/-^ and incubated with DMEM containing 200 μM odorant or 0.1% DMSO (control) at 37°C for 48 h. The cell layers were monitored at 0 h, 24 h and 48 h and documented using a digital camera. The overgrowing gap was measured, and the gap area was calculated relative to the initial scratch area (0 h).

### Caspase assay

The Caspase-3 Colorimetric Assay Kit was purchased by Abcam and immunocytochemical staining was performed according to the manufacturer’s protocol. HCT116 cells were seeded on 12-mm coverslips placed in 24-well plates, filled with medium and grown overnight. Cells were treated with Troenan (200 μM), DMSO and medium (serving as control) for 24 hours. 4%-PFA was used to fix the cells for 20 min and cells were washed twice with 2 ml PBS+Triton X-100 for 5 minutes and one time for 15 minutes. Cells were blocked with blocking buffer (PBS + 10% fish gelatin + 0.5% goat serum) for 1 hour at room temperature. Incubation with the primary antibody detecting caspase-3 (diluted 1:300 in blocking solution) was performed overnight at 4°C. Monoclonal primary antibody (rabbit anti-caspase-3) was purchased from Alomone. Cells were washed with PBS+TritonX-100 3 times for 10 minutes and incubated with the second antibody (purchased from Cell Signaling) 1:1000 including 2 μl DAPI (4´,6-diamidino-2-phenylindole). After 1 hour of incubation at room temperature, cells were fixed with ProLong® Gold Antifade (Life Technologies) and analyzed with an LSM 510 Meta confocal microscope (Carl Zeiss, Oberkochen, Germany)

### Phalloidin staining

For staining of the filamentous actin, Alexa Fluor 488 ® by Thermo Fisher was used. Staining was performed according to the manufacturer’s protocol. In short: Cells were seeded coverslips placed in 24-well plates, filled with medium and grown overnight. Then, cells were stimulated with Troenan and DMSO as a control for 24 hours. On the next day, cells were washed with PBST + 0.1% Triton for 5 minutes. Staining solution was added on the coverslip for 20 minutes at room temperature. Cells were washed twice with PBS and analyzed with an LSM 510 Meta confocal microscope (Carl Zeiss, Oberkochen, Germany).

### Serotonin assay

HCT116 cells were seeded in 96-well plates, filled with medium and grown overnight. Then, cells were washed twice with PBST and incubated with Ringer’s solution containing Fluoxetine (2 μM), Troenan (700 μM) or DMSO for 1 hour. 5-HT levels were measured by using a commercial enzyme immunoassay kit according to the manufacturer’s protocol (5-HT ELISA, Enzo Life Sciences AH Diagnostic AB, Solna, Sweden).

### Western blot

HCT116 cells were treated with Troenan (200–300 μM) for 15 and 25 min. After the incubation, cells were trypsinized, suspended and homogenized in lysis buffer (50 mM Tris/HCl, pH 7.4, 150 mM NaCl, 1 mM EDTA, and 1% Triton X-100), containing PhosSTOP Phosphatase inhibitor Cocktail Tablets (Roche, Basel, Switzerland) and Complete^TM^ Protease Inhibitor Cocktail Tablets (Roche). Protein concentration of the supernatant -containing whole cell lysate—was determined as described [30]. Western blots were performed as previously described [18]. Signals were detected with the ECL^TM^ Select Western Blotting Detection System (Amersham Biosciences, GE Healthcare, Solingen, Germany). The following antibodies were used: phospho-PKC_δ_, phospho-PKC_θ_, p38, phospho-p38, Akt, phospho-Akt (T308), ERK, phospho-ERK, phospho-Fyn, phospho-mTor, SAPK, phospho-Stat2, Stat3, phospho-Stat3, Stat5 and phospho-Stat5. An antibody for β-actin served as a control.

### shRNA preparation, lentivirus production and lentiviral infection

A puromycin-sensitive pLKO vector, containing a 1.8 kb stuffer sequence in place of the shRNA cassette, was used to construct the cell lines. The stuffer was released by using restriction enzymes, and the annealed shRNA, containing both oligonucleotides (sense/antisense), was inserted into the vector. This ligation mixture was used to transform competent cells using a standard electroporation procedure. The transformed cells were plated on ampicillin-containing agar plates, and the over-night cultures of individual colonies were collected and prepared by using the Pure Yield Plasmid MidiPrep System (Promega, Mannheim, Germany) following the manufacturer’s protocol. Lentiviruses were produced as described [31]. The transfection rate was controlled by a parallel infection under identical conditions using a lentiviral GFP expression control vector (pRRLU6-CPPT-pSK-GFP). 6 days after infection 1 μg/ml puromycin (Life Technologies) was added to the cell culture media to select the cells containing the shRNA cassette.

pLKO-shRNA-OR vectors: 4 pLKO-shRNA-OR vectors targeting different regions of the OR were constructed by annealing the sense and antisense oligonucleotides to each other. The resulting double-stranded DNA molecule was cloned into the AgeI/EcoRI site of the pLKO-puro vector (kindly provided by Sheila Stewart). The following oligonucleotides were used for cloning: pLKO-shRNA-OR-402:

sense: 5´-CCGGCATTGTAGAATCAGGTATCTTCTCGAGAAGATACCTGATTCTACAATGTTTTTG-3’,

antisense: 5´- AATTCAAAAACATTGTAGAATCAGGTATCTTCTCGAGAAGATACCTGATTCTACAATG-3´

shRNA-vector preparation, lentiviral production and infection were performed as described (31). For the HCT116 cell line, a stable control cell line, containing an empty lentiviral vector was established. 4 different vector constructs were designed to create the knock down cell line. The most efficient cell line was HCT116-sh-10F1, and this was used for further studies. A Tet-On gene expression system was used to induce doxycyclin-controlled transcription of the shRNA, which knocks down OR51B4. For further information see S5.

### Statistics

Results were tested for normality (Shapiro-Wilk) and equal variance. For normally distributed data, we used a two-tailed unpaired Student´s *t*-test. A Mann-Whitney Test was performed for data that were not normally distributed. If not stated otherwise, every result contains the data from three independent experiments, so the SEM (standard error of the mean) is shown. The statistical significance is indicated as follows: *p < 0.05, **p < 0.01, ***p < 0.001.

## Results

### Ectopic expression of ORs in colon cancer cells and native tissues

**Fig 1:**
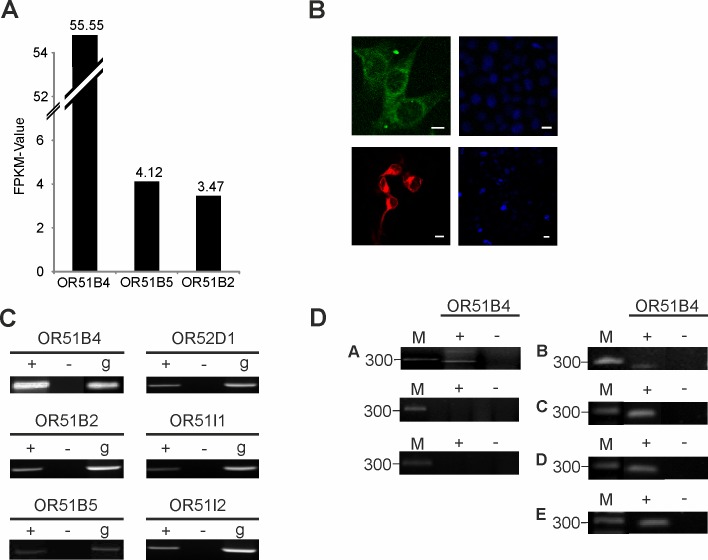
Expression of OR51B4 in HCT116 cells and colon cancer tissues. **(A)** Bar chart displays the FPKM values of the most highly expressed ORs in HCT116 cells. **(B)** Immunocytochemical staining of OR51B4 in HCT116 cells with a specific OR51B4-antibody. Left: HCT116 cells. Right: Negative control: HCT116 cells stained with second antibody alone. Bottom left: Hana3A cells transfected with OR51B4 plasmid. Bottom right: untransfected Hana3A cells. Scale bar: 10 μm. **(C)** The most highly expressed ORs in NGS analyses, validated by RT-PCR. + = +RT, cDNA;— = -RT, RNA; g = genomic DNA as a control; M = marker. **(D)** OR51B4 expressed in human colon cancer tissues: RT-PCR analysis shows OR51B4 expression in human colon cancer tissues. Expression in A: Colon tissue B: Colorectal cancer tissue C, D, E: Colon carcinoma tissues. M = marker.

To identify odorant receptors showing ectopic expression in colon cancer cells, we analyzed RNA-Seq transcriptome data from the colon cancer cell line HCT116. The Illumina RNA-Seq protocol was used to generate up to 5 million reads per transcriptome, with a read length of 101 bp. By focusing on the ectopic expression of ORs, we detected mRNA transcripts from 3 different ORs with FPKM-value > 3.0 (Fig 1A). A ranking of these receptors by expression level showed the highest expression for OR51B4, with an FPKM-value of 56. Next, we analyzed published transcriptome data for the HCT116 cell line in the same way to validate the high expression of OR51B4. Exceptionally high expression of OR51B4 (average FPKM-value: 25.9) was confirmed in the other RNA-Seq data sets (S1C Fig). All ORs were ranked by expression level (S1A Fig). To validate our sequencing data, we analyzed the distribution of mapped reads for OR51B4 using the Integrative Genomic Viewer (www.broadinstitute.org/igv) (S1B Fig). On average, 22 ORs are expressed in HCT116 cells with an average FPKM-value of 2.5 (without OR51B4) and 3.79 (with OR51B4). NGS data were confirmed by RT-PCR experiments (Fig 1C). Furthermore, we validated the expression of OR51B4 in HCT116 cells at the protein level by conducting immunocytochemical staining with a specific OR51B4 antibody (Fig 1B). We validated its specificity by immunocytochemical staining of Hana3A cells transfected with the OR51B4 expression plasmid (Fig 1B). The OR51B4 protein was located in the cell membrane and in vesicular structures within the cell. As HCT116 serves as a model system for colorectal cancer, we investigated the expression of OR51B4 in several human cancer tissues. OR51B4 was detected in tissue samples of rectal and colon carcinoma (Fig 1D). However, its level of expression might be donor-specific. Nevertheless, these findings underline the possible clinical relevance of the receptor.

### Deorphanization of OR51B4

**Fig 2:**
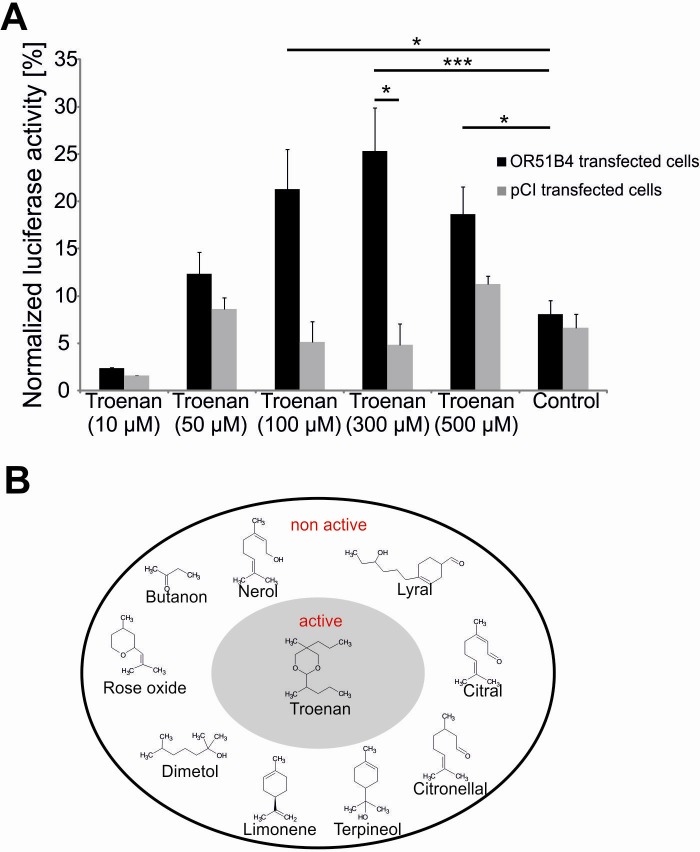
Deorphanization of OR51B4. **(A)** Activation of OR51B4 transfected Hana3A cells by Troenan. Bar chart shows luminescence values upon activation. Luminescence values are normalized to Forskolin activation. OR-transfected cells: Hana3A cells transfected with pCI-vector containing OR51B4. pCI-transfected cells: Hana3A cells transfected with the pCI-vector alone. Control: cells treated with DMSO (0.1%) **(B)** Molecular receptive field of recombinant expressed OR51B4. Grey inner circle: agonist, white outer circle: inactive substances. N > 3 independent CRE-Luciferase assays.

As OR51B4 is an orphan receptor, we first performed deorphanization experiments for the receptor in a CRE-Luciferase reporter gene assay. We used Hana3A cells [27],—stably expressing several accessory proteins for the olfactory signaling cascade—to identify an activating ligand. Using the CRE-Luciferase reporter gene assay, we screened the Henkel100 library, containing 100 structurally different substances [29], to identify specific ligands. The experiments revealed significant cell activation by 5-methyl-5-propyl-2-(1-methylbutyl)-1,3-dioxane, also known as Troenan. Troenan significantly increased the cAMP level in the CRE-Luciferase assay (Fig 2A). There is slight background activation in pCI-transfected cells, however it is regardless of the Troenan stimulation, as it occurs in control cells as well. Troenan in concentrations >100 μM elicits significant luciferase activity and therefore, it can be considered to be a ligand for OR51B4. Several other substances did not evoke cell activation (Fig 2B).

### Characterization of HCT116 cell activation upon Troenan stimulation

**Fig 3:**
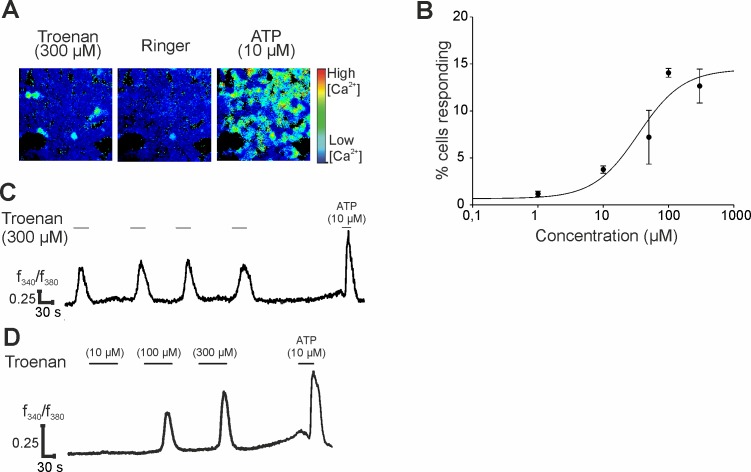
Characterization of Troenan-induced calcium signals in HCT116 cells. **(A)** Representative image of HCT116 cells stimulated with Troenan (300 μM) in calcium imaging analysis. **(B)** Number of cells responding to Troenan in different concentrations. **(C)** Repetitive activation of HCT116 cells upon repetitive Troenan application. **(D)** Dose-dependent activation of HCT116 cells by Troenan. Troenan was applied at concentrations of 50 μM, 100 μM and 300 μM. Peak amplitudes show the increases in intracellular calcium concentration. To ensure viability of the cells, ATP was applied last, which serves as a positive control. N > 3 with n = 18 measurements in 9 cell culture dishes with approximately 200 cells.

The activation of HCT116 cells upon Troenan stimulation was further analyzed by calcium imaging (Fig 3A). We were able to show that, with increasing Troenan concentration, the number of activated cells increases (Fig 3B). This was also in accordance with dose-dependent increasing amplitudes based on the calcium influx into the cells (Fig 3C and 3D). Up to 13% of all HCT116 cells were activated by 300 μM Troenan. At higher concentrations, the number decreased (Fig 3B). Application of Troenan leads to a transient increase in the intracellular calcium concentration of HCT116 cells (Fig 3C). To monitor the long-term effects of Troenan stimulation (100 μM) on OR51B4-expressing HCT116 cells, Troenan was applied for 20 minutes and was used to induce repetitive calcium responses in the cells (S2 Fig).

### Specificity of OR51B4-expressing cells

**Fig 4:**
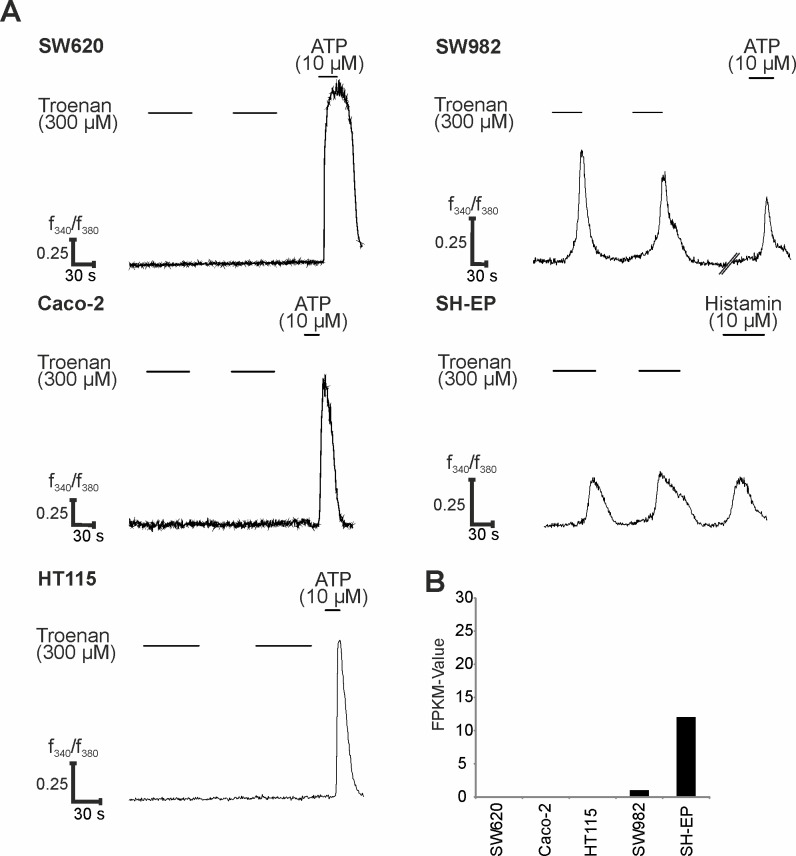
Effect of Troenan application on different cancer cell lines. **(A)** Calcium signals upon Troenan application (300 μM) in the cancer cell lines SW620, Caco-2, HT115, SW982 and SH-EP. N = 3 with n = 6 measurements in 3 cell culture dishes with approximately 200 cells. **(B)** RNA-Seq data analysis for all cell lines. Bar chart showing the FPKM values in five different cell lines.

In a broad analysis of NGS-based transcriptome data from different carcinoma cell lines, we detected other cell lines expressing OR51B4 at the transcript level: SW982, a sarcoma cell line, and SH-EP, a neuroblastoma cell line. We analyzed the correlation between Troenan-induced calcium responses and OR51B4 expression. Cell lines that showed no OR51B4 expression (Caco-2, HT115 and SW620) elicited no calcium signals upon Troenan application in calcium imaging experiments. In contrast, OR51B4 was expressed with FPKM-values of approximately 1 and 12 (Fig 4B) in the cell lines SW982 and SH-EP, respectively. In our experiments, the application of Troenan evoked intracellular Ca^2+^ increases, suggesting specific activation of OR51B4 by this ligand (Fig 4A). In SH-EP and SW982 cells, up to 5% of the cells responded to Troenan.

### Characterization of the signaling pathway

**Fig 5:**
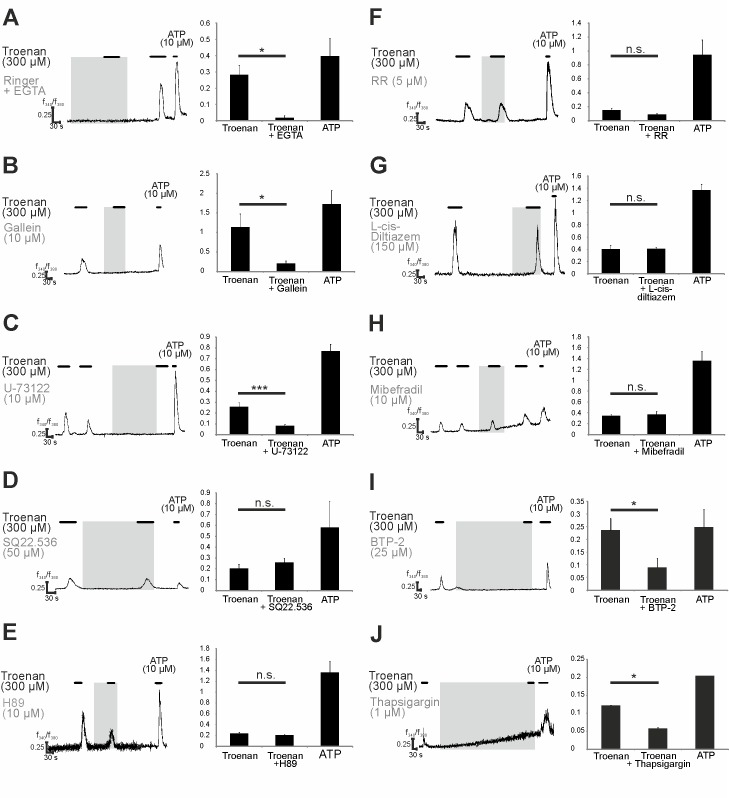
Pharmacological analysis of the signaling pathway involved in the activation of OR51B4. Representative calcium imaging traces of HCT116 cells stimulated with Troenan and different specific inhibitors. Grey area represents the duration of the inhibitor applicated. Bar chart showing mean amplitudes of Troenan-induced Ca^2+^ signals in HCT116 cells. **(A)** Localization of Ca^2+^ by use of EGTA-Ringer. Investigation of different specific inhibitors of calcium signaling upon Troenan stimulation. (**B**) Gallein (10 μM), **(C)** U-73522 (10 μM), **(D)** SQ22.536 (50 μM), **(E)** H89 (10 μM), **(F)** RR; Ruthenium red (5 μM), **(G)** L-cis-diltiazem (150 μM), **(H)** Mibefradil (10 μM), **(I)** BTP-2 (25 μM), **(J)** Thapsigargin (1 μM). N > 3 with n = 18 measurements in 9 cell culture dishes with approximately 200 cells. The data are shown as the mean SEM.

To characterize the signal transduction pathway downstream of the OR51B4 activation, we conducted different calcium imaging experiments, including measurements using specific pathway inhibitors. First, we determined the source of the calcium involved in the activation of the cells. Measurements under calcium-free conditions revealed that the Troenan-induced responses depend on extracellular calcium (Fig 5A). Then, we analyzed the influence of the specific Adenylate Cyclase (AC)3-inhibitor SQ22.536, which did not affect the calcium responses (Fig 5D). To determine the G-Protein subunit involved, we used Gallein–an inhibitor of G-protein subunit βγ –and we showed significant inhibition of Troenan-induced calcium signals, indicating involvement of the G-Protein subunit βγ (Fig 5B). Gβγ is capable of activating Phospholipase C, so its involvement was investigated by using the inhibitor U-73522, which significantly inhibited the Troenan-induced calcium signals (Fig 5C). Because Phosphokinase A (PKA) can be directly activated by a G-protein, the use of H89, an inhibitor of PKA, validated that the signal transduction is independent of PKA (Fig 5E). In further studies, we tried to identify a possible effector channel mediating the calcium influx. Neither T-Type calcium channels, nor the cyclic nucleotide-gated (CNG) channels or transient receptor potential (TRP) channels were involved, which was validated by the ineffectiveness of the specific inhibitors Mibefradil (Fig 5H), L-cis-diltiazem (Fig 5G) and Ruthenium red (Fig 5F). Blocking the Troenan-responses by using the inhibitor BTP-2 revealed that store-operated calcium channels can serve as effector channels. Thus, we used the inhibitor BTP-2, which inhibits calcium influx via store-operated channels (Fig 5I). To ensure this mechanism, we analyzed the involvement of internal calcium stores by inhibiting the sarcoplasmatic reticulum Ca^2+^ ATPase with Thapsigargin. After store depletion by Thapsigargin, Troenan failed to induce calcium signals in HCT116 cells (Fig 5J).

**Fig 6:**
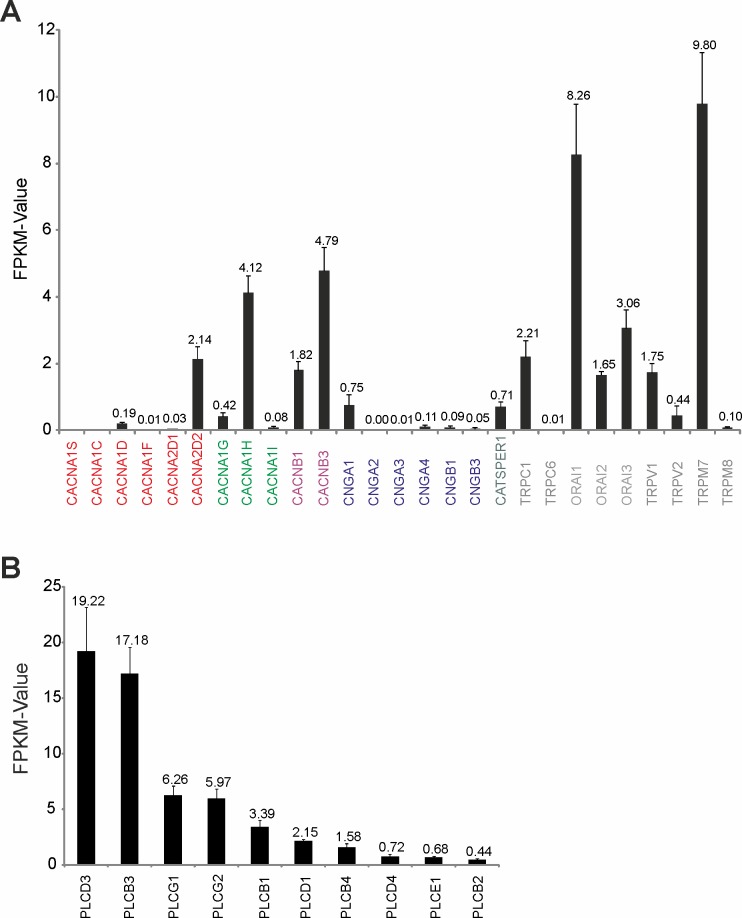
Transcript abundance of potential effector channels in HCT116 cells determined by RNA-Seq. **(A)** Bar chart showing the FPKM values of different possible effector channels. Voltage-dependent L- and T-Type channels: CACNA1S, CACNA1C, CACNA1D, CACNA1F, CACNA2D1, CACNA2D2; CACNA1G, CACNA1H, CACNA1I; CACNB1, CACNB3. Cyclic nucleotide-gated ion channels: CNGA1, CNGA2, CNGA3, CNGA4, CNGB1, CNGB3. Voltage-dependent Ca^2+^ channel: CATSPER1. Transient receptor potential channels: TRPCI, TRPC6, TRPV1, TRPM8, TRPC6, TRPV2, TRPM7, TRPM8. Calcium release-activated calcium channels: ORAI1, ORAI2, ORAI3. **(B)** Transcript expression of PLC isoforms in the colon cancer cell line HCT116. Bar chart showing FPKM values of different PLCs in the colon cancer cell line HCT116.

The analysis of the signaling pathway via calcium imaging experiments revealed initial insights into the different components involved in Troenan stimulation. We next analyzed the RNA-Seq data of the cell line with a focus on the signaling cascade components. CNG-channels and the CatSper channel show only low expression rates (Fig 6A). The expression levels of TRP-channels vary from FPKM 0.1 (TRPM8) to 9.8 (TRPM7). ORAI1, 2 and 3 are expressed, which are additional indications that these channels serve as effector channels. The involvement of PLC, as observed by inhibition with U-73522, can be affirmed by transcriptome data as well (Fig 6B). We also confirmed the expression of Phospholipase C (PLC) via RT-PCR (S3 Fig). Thus, the existence of these components at the transcriptome level is in accordance with the inhibitor experiments.

**Fig 7:**
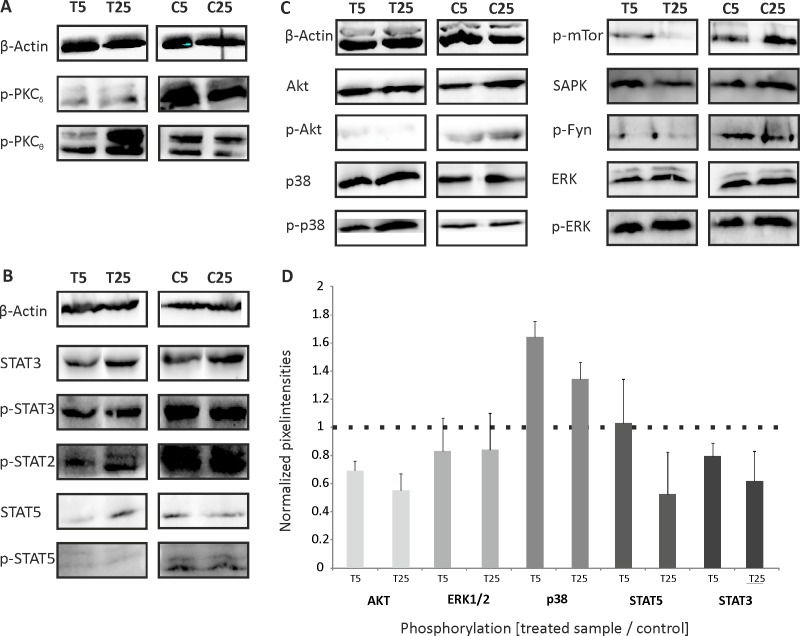
Western blot analysis of HCT116 cells stimulated with Troenan (300 μM; T) or control (C) for 5 and 25 minutes. **(A)** Phosphorylation of different isoforms of PKC. **(B)** Reduced phosphorylation of Stat2, Stat3 and Stat5 upon Troenan stimulation (300 μM). **(C)** Stimulation of Troenan (300 μM) leads to the time-dependent phosphorylation of p38 MAPK. Reduced phosphorylation was observed for Akt, mTor and Fyn. Troenan stimulation did not affect ERK and SAPK. The total amounts of p38, Akt, ERK, Stat3 and Stat5 and β-actin served as controls. n = 3. **(D)** Quantification of the mean pixel intensities of the phosphorylated protein kinases Akt, ERK1/2, p38, Stat5 and Stat3. The pixel intensities of duplicates were averaged. Total amounts of the protein kinases were determined and served as controls.

To study the participation of protein kinases in downstream signaling, we monitored the phosphorylation of different kinases and targets that are crucially involved in cellular processes associated with cancer by using Western blot experiments. We tested whether Troenan alters the phosphorylation status of diverse targets. HCT116 cells were exposed to Troenan (300 μM) for 5 and 25 minutes (Fig 7). PLC is capable of activating proteinkinase C (PKC), therefore, we analyzed the phosphorylation of PKC_Θ_ and PKC_δ_ (Fig 7A). Here, we investigated a reduced phosphorylation of p-PKC_δ_ and a time-dependent increase in phosphorylation of p-PKC_Θ_. Several Stat-proteins show a reduced phosphorylation upon Troenan stimulation. The same applies to Akt, a well-known component needed for cell survival, whereas p38 is phosphorylated (Fig 7C). Furthermore, phosphorylation of mTor and Fyn are reduced. No effects were observed on ERK and SAPK.

### Physiological effects of Troenan stimulation on HCT116 cells

**Fig 8:**
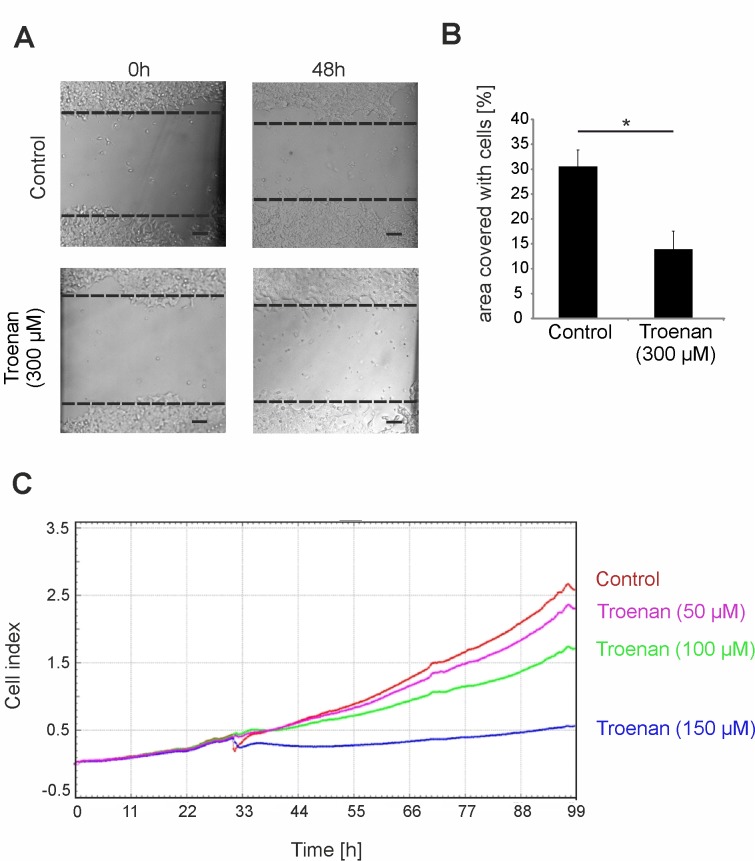
Physiological effects of Troenan stimulation on HCT116 cells. **(A)** Analyses of cell migration by scratch assay after Troenan stimulation (300 μM) for 48 hours. **(B)** Bar chart showing statistical analysis of the area overgrown in scratch assay experiments. n = 3 assays. **(C)** Monitoring of the cell-index equal to the cell proliferation rate of HCT116 cells incubated with Troenan in different concentrations (50, 100, 150 μM). Dynamic real-time monitoring of cell processes in vivo (xCELLigence RTCA-technology). n = 2 in at least 2 independent experiments.

After demonstrating a Troenan-mediated calcium signal in HCT116 cells, we investigated the effect of Troenan stimulation on cell physiology. We analyzed the effect on cell migration by using scratch assays. Cells were incubated with Troenan (300 μM) for 48 hours and we analyzed the area covered with cells by the software *tscratch*. There was a significant reduction in cell migration in the cells incubated with Troenan compared to untreated cells (Fig 8A and 8B). Furthermore, we conducted analysis of cell proliferation using xCELLigence RTCA-technology, which allows label-free real-time monitoring of cell viability and proliferation (Fig 8C). Stimulation of HCT116 cells for 24 hours caused a reduction in cell number and induced morphological changes, such as impaired colony formation.

**Fig 9:**
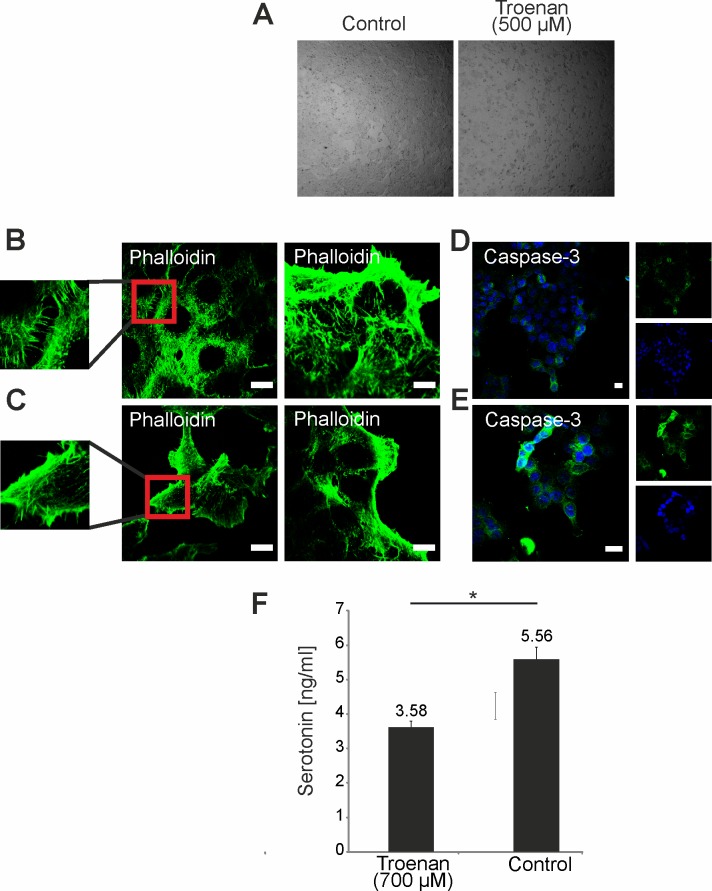
Impaired actin filament formation and induction of apoptosis upon stimulation of HCT116 cells with Troenan. **(A)** HCT116 cells treated with Troenan (500 μM) and control cells. **(B) and (C)** Phalloidin staining of control cells (B) and cells treated with Troenan (300 μM) (C). Scale bar: 10 μm. **(D) and (E)** Immunocytochemical staining of HCT116 cells with an antibody against caspase-3 after treatment with control (D) or Troenan (300 μM) (E). Cells treated with Troenan (300 μM) for 48 hours. Scale bar: 10 μm. **(F)** HCT116 cells show decreased serotonin release after application of Troenan (700 μM) for 60 minutes.

After showing that Troenan stimulation leads to impairment of cell migration, we analyzed changes in cell morphology in general (Fig 9A) and particularly in actin filament polymerization upon ligand application (Fig 9B). We observed a reduction of cellular protrusions (Fig 9B). Next, we performed immunocytochemical staining with a caspase-3-specific antibody in HCT116 cells. Caspase-3 positive immuno-detection suggests the induction of apoptosis upon Troenan stimulation. Here, we demonstrated that HCT116 cells treated with Troenan show distinct caspase-3 staining compared to control cells (Fig 9D and 9E). Several disorders of the gastrointestinal tract are associated with dysregulated serotonin signaling. Approximately 95% of the body’s serotonin stores are found in the gastrointestinal tract [32]. Therefore, we measured the serotonin amount in the supernatant of the cells after stimulation with Troenan (700 μM) for 60 minutes. Interestingly, Troenan stimulation led to a significant decrease in serotonin concentration in the supernatant (Fig 9F).

### Troenan-induced Ca^2+^ signals are mediated by OR51B4

**Fig 10:**
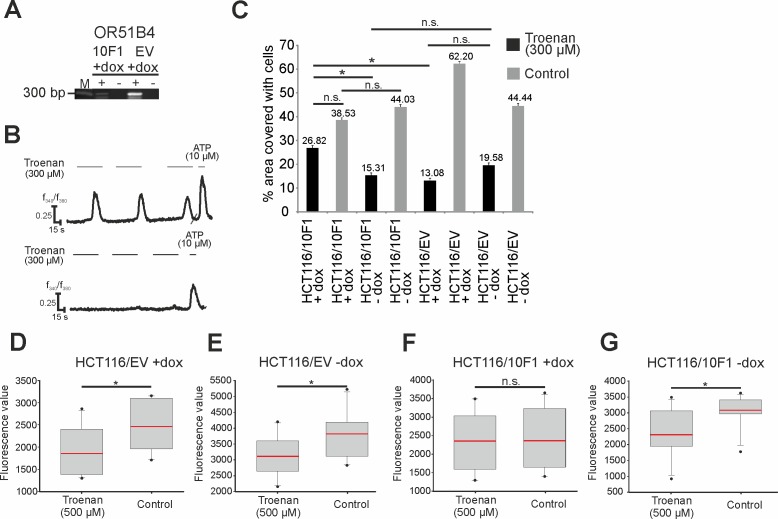
Analysis of the Troenan-induced effect in HCT116 cells containing a doxycycline-sensitive OR51B4-knockdown-sequence. **(A)** Confirmation of knockdown functionality by qRT-PCR and calcium imaging experiments. M = Marker. Stimulation of HCT116/EV (left) and HCT116/10F1 cells (right) with Troenan (100 μM/ 300 μM). **(B)** Representative calcium signal of HCT116/EV (above) and HCT116/10F1 (below) cells stimulated with Troenan (300 μM) in calcium imaging analysis. **(C)** Migration analysis via scratch assay with HCT116/EV and HCT116/10F1 cells with and without doxycycline induction. Stimulation of the cells with Troenan (300 μM) for 48 hours. N = 3 assays with 3 dishes. **(D)-(G)** Proliferation analysis of HCT116/EV (D, E) and HCT116/10F1 (F, G) cells after treatment with Troenan (300 μM) with and without doxycycline induction. Troenan (300 μM) was applied for 72 hours. N = 20.

To prove that Troenan-induced signals are evoked by the participation of OR51B4, we generated cells with a knockdown of the OR51B4 gene. Using the ΔΔCT-method, we detected a 5-fold decrease in OR51B4, which is expressed under a doxycycline-sensitive promoter, as shown by real-time PCR experiments (Fig 10A). Troenan (300 μM) application in calcium imaging experiments evoked almost no calcium signals in HCT116-knockdown cells (Fig 10B).

After validating the successful knockdown of OR51B4 by the use of doxycyclin, we examined the physiological effects of Troenan exposure via scratch assays. Troenan stimulation in OR51B4 positive cells significantly reduced the cell migration (Fig 10C). In contrast, the cell migration of Troenan-exposed knockdown cells (via doxycycline induction) was equal to that of control cells (exposed to DMSO). Furthermore, we conducted proliferation assays and analyzed the effect of Troenan stimulation on cells with and without doxycycline (Fig 10D–10G). Obviously, there was significantly reduced proliferation in Troenan-stimulated cells in comparison to control cells, but there was no difference between doxycycline-induction and control treatment in the control cell line HCT116/EV (Fig 10D and 10E). The induction of the knockdown in HCT116/10F1 cells by doxycycline lead to abolishment of the difference, so that Troenan-treated and control cells showed similar proliferation rates (Fig 10F). In HTC116/10F1 cells without doxycycline induction, the difference in the proliferation rates persisted (Fig 10G).

### Expression profile of OR51B4 in different carcinoma tissues and cell lines

**Fig 11:**
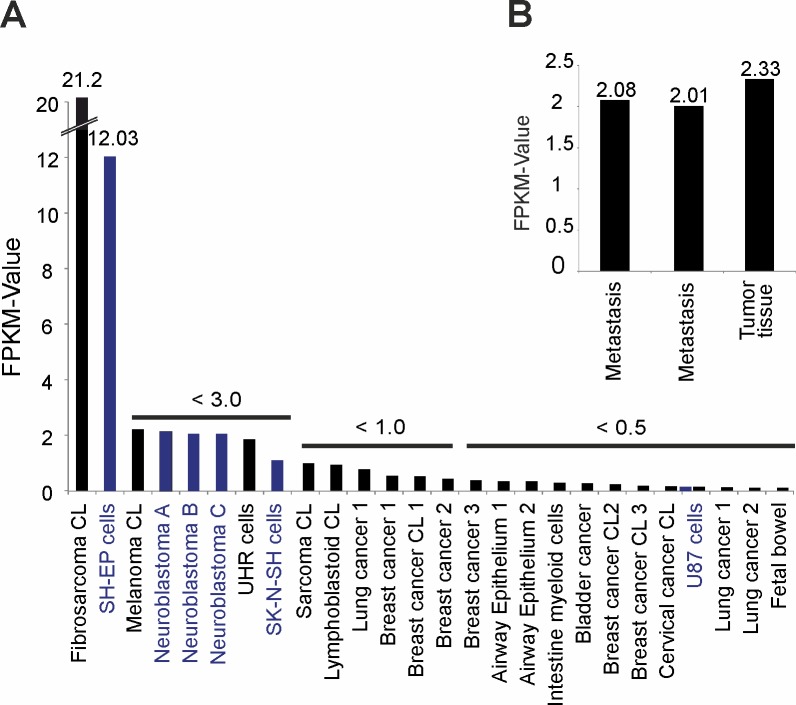
Expression profile of OR51B4 in different carcinoma tissues and–cell lines. **(A)** Bar chart showing the ranked FPKM values obtained from RNA-Seq data for other tissues and cell lines. **(B)** Expression of OR51B4 in the transcriptomes of different colon cancer tumors and metastatic tumors.

To determine the expression profile of OR51B4, we analyzed more cancer tissues and cell lines and produced a ranking of the expression values of OR51B4 in the different tissues and cell lines. The fibrosarcoma cell line—HT1080—displays the highest expression for OR51B4 (FPKM value: 21.2) (Fig 11A). The second-highest expression was found in a neuroblastoma cell line (SH-EP, FPKM value: 12). OR51B4 is also expressed in some lung-, breast-, bladder- and cervical cancer tissues, leading to the assumption that this receptor might play a role in several different cancers.

Interestingly, OR51B4 is also expressed in several human neuroblastoma tissues and glioblastoma cell lines, e.g., SHEP, SK-N-SH and U87 (Fig 11A, blue). Among them, SH-EP cells show the highest expression. We also analyzed the transcriptomes of patients with colon carcinomas and liver metastases, which revealed the expression of OR51B4 in 3 out of 4 data sets (Fig 11B). These results demonstrate that the carcinoma-specific expression and distribution of OR51B4 reveal the regulatory importance of this receptor.

## Discussion

Several studies have successfully demonstrated the expression of ORs at the mRNA level in various human tumor tissues [14,18,20,21] but less is known about the function of these ORs. However, the presence in tumor tissue implies the potential function for ORs to be used as novel targets in cancer therapy.

In this study, for the first time, we demonstrate the ectopic expression of the olfactory receptor OR51B4 in the colon cancer cell line HCT116 and reveal the influence of its activation by the cognate ligand Troenan on cell proliferation, migration, actin filament formation and apoptosis.

We detected OR51B4 mRNA in HCT116 cells at a high expression level (FPKM: 56) via NGS analyses. The data were validated in 5 different data sets, all consistently confirming the high expression of OR51B4. NGS data could be corroborated by RT-PCR analysis. The high mRNA level and the presence of this receptor at the protein level in HCT116 suggest a functional role. Because the expression of OR51B4 is not restricted to HCT116, but rather is also present in two other colon cancer cell lines and is even expressed in native colon carcinoma tissues, the potential importance of OR51B4 for clinical research and treatment of colon cancer is underlined. In order to analyze the function of OR51B4, we first of all had to identify the ligand by reporter gene assay-based deorphanization studies, which revealed Troenan (5-methyl-2-pentan-2-yl-5-propyl-1,3-dioxane) as a ligand. Troenan has a watery green floral odor. It is capable of inducing transient intracellular Ca^2+^ elevation in HCT116 cells. That elevation of cytosolic Ca^2+^ in HCT116 cells upon Troenan stimulation is mediated by OR51B4, as was successfully demonstrated by shRNA knockdown experiments. However, there may be unknown endogenous ligands for OR51B4, which was already demonstrated for OR51E2 in prostate cells [18], that were activated by the odorant β-ionone and by 6-dehydrotestosterone as an endogenous ligand.

There is large body of evidence that odorants, especially terpenes, can affect the viability and cell biology of cancer cells, even in colon cancer [33]. Some odors can induce the phosphorylation of AMP-activated protein kinase (AMPK), leading to cell cycle arrest and apoptosis in a colon cancer cell line [34]. Linalool is capable of mediating antiproliferative effects in melanoma cells [35]. However, both studies failed to identify any of the molecular mechanisms responsible for these effects.

We showed that Troenan stimulation of cancer cells induces significant changes in cell development, colony formation and viability. We recognized at first glance that incubation with Troenan leads to obvious morphological changes in cell shape and causes impaired colony formation. The pathway involving Akt is capable of regulating actin, the modeling of which is associated with the reorganization of the actin cytoskeleton and thus is associated with cell migration [36]. Furthermore, a study by Zhang et al. showed the influence of OR2A4 on the cytoskeleton of HeLa cells [37]. We observed inhibition of the filopodial protrusion and impaired formation of multicellular spheroids upon Troenan stimulation. Because the burden of metastasis contributes in large part to the morbidity and mortality of colorectal cancer [38], we analyzed the effects of Troenan treatment on cell migration via scratch assays. Here, we showed that Troenan stimulation leads to significantly reduced cell migration. Thus, we proved that the effect is mediated by OR51B4, because, as in HCT116-knockdown cells, the reduced migration was diminished upon Troenan stimulation. We also analyzed the effect of Troenan treatment on cell growth and proliferation. Our data demonstrate that cell growth and proliferation is altered upon Troenan stimulation. The significant dose-dependent reductions in cell proliferation after long-term treatment of HCT116 cells demonstrate the pharmacological potential of this receptor. These results are in accordance with studies showing that OR activation via Citronellol or β-ionone similarly lead to reduced proliferation of hepatocarcinoma and prostate cancer cells [14,18].

Interestingly, Serotonin plays a fundamental role in tumor growth and differentiation and acts as a growth factor for several tumor types [39]. Several studies have demonstrated the mitogenic effects of serotonin receptors in prostate, bladder and breast cancer. It has been shown that 5HT might have mitogenic roles in colorectal cancer [40]. Here, we showed that the application of Troenan leads to decreased serotonin release in HCT116 cells. The effects of Troenan on the cells correspond to the findings of Ataee et al., showing an anti-mitogenic effect of a 5HT antagonist [40].

We showed that OR51B4-Troenan interaction leads to changes in the intracellular Ca^2+^ levels and aimed to elucidate the underlying signaling pathway responsible for the physiological changes. Therefore, we performed pharmacological experiments using specific inhibitors: Inhibitors for components of the canonical pathway did not affect OR51B4-mediated calcium signals, which is not unusual, as the transduction mechanism of ORs is highly variable and can involve PLC via IP_3_ [41,42]. Therefore, we used a PLC-inhibitor and confirmed the involvement of PLC upon Troenan stimulation. We demonstrated that PLC activation is mediated via βγ-subunit of the G-protein, which was also shown by Smrcka et al. [43].

In order to identify the effector channel mediating the calcium influx, we analyzed the involvement of different calcium channels and excluded the involvement of TRP-channels, some types of T-Type calcium channels and CatSper channels. In several cancer types, calcium channels involved in calcium signaling pathways promoting cancer behaviors are mostly non-voltage activated and belong to the TRP or ORAI superfamily [44]. Interestingly, store-operated Ca^2+^ entry (SOCE) is the predominant Ca^2+^ entry mechanism in most cancer cells [45], so we used a CRAC channel inhibitor that revealed the involvement of these channels. The complete inhibition of calcium signals after incubation with Thapsigargin, an inhibitor of the sarco/endoplasmatic reticulum Ca^2+^ ATPase, confirmed ORAI channels as effector channels. In addition, we analyzed the expression levels of the different signaling pathway components implicated by the NGS-based transcriptome data. Interestingly, the NGS-based transcriptome data were in accordance with our pharmacological studies as the experimental data correlate with the mRNA levels of the signaling pathway components.

Further downstream of the signaling cascade, Troenan stimulation might lead to the phosphorylation of MAPKs, mediating the reduction of proliferation and induction of apoptosis, which was already demonstrated in hepatocarcinoma cells and leukemia cells stimulated by citronellal and sandalore [14,46].

**Fig 12:**
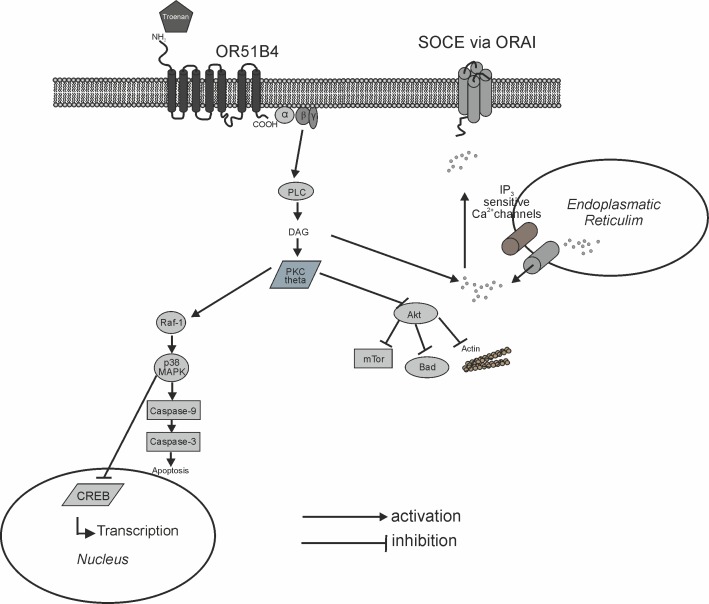
Diagram of the proposed signaling cascade that is induced by Troenan stimulation. PLC: phospholipase C, DAG: diacylglycerol, PKC: protein kinase C, CREB: cAMP response element-binding protein, SOCE: store-operated calcium entry.

Thus, we concentrated on the phosphorylation of kinases often involved in cancer signaling pathways. Western blots revealed that Troenan stimulation leads to phosphorylation of p38 mitogen-activated protein kinase, possibly mediated by PKC_θ,_ a key molecule in antitumor immune surveillance [47], which was also found to be phosphorylated. We assume that PKC here is activated by PLC [48] the key component in the Troenan mediated signaling pathway. It is known that p38 MAPK acts as a negative regulator of proliferation in colon cancer cells and furthermore is capable of transducing apoptotic stimuli [49]. The key molecule for cell survival, Akt, is considered to be a target for cancer therapies, as several human cancers show an overexpression of Akt [50]. Here, Troenan stimulation led to a reduced phosphorylation of Akt in a time-dependent manner. The same applies to mTor, which also shows a reduced phosphorylation upon Troenan stimulation. Both the significant reduction of phosphorylation of Akt and mTor are indicators of apoptosis. Apart from that, our results indicate negatively regulated proliferation processes due to the activation of p38 MAPK, which was also shown in LNCaP-cells [18]. The proposed signaling pathway is illustrated in Fig 12.

For the first time, we have demonstrated the OR51B4-mediated anti-proliferative, pro-apoptotic and serotonin diminishing effects of Troenan in HCT116 cells. We hypothesize that these findings will contribute to research on therapies for colon carcinoma and might give way to further investigation of alternative tumor targets. Because colon tumors are often accessible from the lumen, oral application of Troenan is conceivable. This underlines the importance of ectopically expressed ORs for scientific research on cancer pathogenesis and the development of new tumor targets.

## Supporting information

S1 Text Reverse transcriptase PCR (RT-PCR). The mixture contained 10 μl of GoTaq qPCR Master mix, 50 pmol Primers (fwd+rev) and cDNA (equivalent of 50 ng of total RNA). The PCR reaction contained the following steps: 3 min at 95°C followed by 40 cycles of 1 min at 59°C (OR51B4) or 60°C (PLC, TBP) and 1 min at 72°C and was performed in the Mastercycler® ep realplex (Eppendorf, Hamburg, Germany). TBP mRNA encoding the TATA-box binding protein was used for relative quantification by the ddCt method.
Primer:
*OR52D1 –forward*: *CCATGCTGGTGAGATTTCCT*
*OR52D1 –reverse*: *GGAGGCACCAGCACATAGAG*
*OR51B2 –forward*: *ACTGGATCTCCATCCCCTTC*
*OR51B2 –reverse*: *AGGGCTTTGGCTCTCTCTTC*
*OR51I1 –forward*: *ATGAGCTTGGATCGCTTTGT*
*OR51I1 –reverse*: *AAGCGGTGAATCATGGAGAC*
*OR51B5 –forward*: *GCAGGAGAGCAAAGAAGTCTC*
*OR51B5 –reverse*: *GGACAGGGGAAGGAGGTAAG*
*OR51B4 –forward*: CGAGAATTCAGCATGTGGTATAACAACAGTGC
*OR51B4 –reverse*: CGAGCGGCCGCGCTTCAAGCCCTACTCTGCCC
*OR51I2 –forward*: *ATGCCCGCAACATCACTT*
*OR51I2 –reverse*: *GCACAGGAGGCACAAATAGG*
*TBP–forward*: *TATAATCCCAAGCGGTTTGC*
*TBP–reverse*: *GCTGGAAAACCCAACTTCTG*
*PLC—forward*: *AGGTTCAGGAGGATGTATGCC*
*PLC–reverse*: *GCTCCTCGAAGTCTGCAGTT*
(DOCX)

S2 Text List of Henkel odorants in Henkel 100. Geranonitrile, acetophenone, eucalyptol, thymol, anethole, menthol, camphor, muscone, citronellol, sweet orange oil, p-cymene, eugenol, geraniol, hedione, helional, lyral, d-carvone,l-carvone, citral a, benzyl acetate, sassafras oil, 15-pentadecanolide, methyl salicylate, linalool, phenylethyl alcohol, hexyl cinnamaldehyde (α), amyl cinnamaldehyde (α), iso-bornyl acetate, dihydro myrcene, benzyl salicylate, galaxolide, oil of turpentine, fixolide np, coumarin, styrlyl acetate, piperonal, jonone (β), ptbca 25 cis, traseolide, aldehyde c12, benzyl benzoate, cyclame aldehyde, dmbca, iso-nonyl acetate, benzophenone, bourgeonal, benzyl alcohol, otbca, cinnamyl alcohol, allyl heptanoate, oxyphenylon, cinnamaldehyde, agrunitril, brahmanol, citrathal, dimetol, epitone, iso-nonyl alcohol, phenylethyl acetate, phenirat, aldehyde c08, ethylfruitat, hexyl acetate, neobergamate, aldehyde c12, anisaldehyde, citrusal, cedryl acetate, ethylvanillin, evernyl, ligustral, dimedone, sandalore, vanillin, aldehyde 11–11, aldehyde 13–13, ambroxan, anthoxan, boisambrene forte, cyclohexyl salicylate, cyclovertal, floramat, herbavert, irotyl, jasmacyclat, melusat, peranat, romilat, sandelice, trivalon, troenan, verdoxan, propidyl, aldehyde c07, alcohol c08, amylbutyrate, prenylacetate, ethylamylketone, methylhexylketone, acedyl.
(DOCX)

S1 Fig Expression level of ORs in HCT116 cells. **(A)** Bar chart showing ranking of OR expression in HCT116 cells. **(B)** Read coverage of OR51B4 detected in HCT116 and visualized by the Integrative Genomic Viewer. **(C)** Average RNA-Seq transcriptome data for OR51B2, OR51B4 and OR51B5 from 5 different data sets.
(TIF)

S2 Fig Long-term application of Troenan (300 μM) on HCT116 cells. HCT116 cells exposed to Troenan (300 μM) for 20 minutes. ATP served as a control.
(TIF)

S3 Fig Expression of signaling pathway components in HCT116 cells validated by RNA-Seq and RT-PCR. ADCY3: adenylyl cyclase 3, GNAQ: G-protein α_q_, GNAL: G-protein α_olf_, GNAI1/3: G-protein α_i_, CNGA1-4: CNG channel subunits (CNGA2, CNGA4, CNGA3, CNGA4, CNGB1 and CNGB3), OMP: olfactory marker protein, ANO2: calcium-activated chloride channel anoctamin 2, REEP1: receptor-enhancing proteins 1, CALM1: Calmodulin 1, RIC8B: nucleotide exchange factor, RTP1: receptor-transporting proteins. PLCγ: phospholipase C γ. PLCβ: phospholipase C β.
(TIF)

## Abbreviations

OR: Olfactory receptor
IHC: Immunocytochemistry
GPCR: G-Protein coupled receptors
PSGR: Prostate-specific G-Protein coupled receptor
PLC: Phospholipase C
FPKM: Fragments per Kilobase of exon per Million fragments mapped
RTCA: Real Time Cell Analysis
DMSO: Dimethylsulfoxid
AC3: Adenylyl cyclase 3
PKA: Proteinkinase A
CNG channel: Cyclic nucleotide-gated channels
TRP channel: Transient receptor potential channel
PKC: Proteinkinase C
