# Supplementary material for: Activation of odorant receptor in colorectal cancer cells leads to inhibition of cell proliferation and apoptosis
Source: PLoS One. 2017 Mar 8;12(3):e0172491. doi: 10.1371/journal.pone.0172491 (PMC5342199; doi:10.1371/journal.pone.0172491)
Supplement: S1 Text — The mixture contained 10 μl of GoTaq qPCR Master mix, 50 pmol Primers (fwd+rev) and cDNA (equivalent of 50 ng of total RNA). The PCR reaction contained the following steps: 3 min at 95°C followed by 40 cycles of 1 min at 59°C (OR51B4) or 60°C (PLC, TBP) and 1 min at 72°C and was performed in the Mastercycler® ep realplex (Eppendorf, Hamburg, Germany). TBP mRNA encoding the TATA-box binding protein was used for relative quantification by the ddCt method. Primer: OR52D1 –forward: CCATGCTGGTGAGATTTCCT OR52D1 –reverse: GGAGGCACCAGCACATAGAG OR51B2 –forward: ACTGGATCTCCATCCCCTTC OR51B2 –reverse: AGGGCTTTGGCTCTCTCTTC OR51I1 –forward: ATGAGCTTGGATCGCTTTGT OR51I1 –reverse: AAGCGGTGAATCATGGAGAC OR51B5 –forward: GCAGGAGAGCAAAGAAGTCTC OR51B5 –reverse: GGACAGGGGAAGGAGGTAAG OR51B4 –forward: CGAGAATTCAGCATGTGGTATAACAACAGTGC OR51B4 –reverse: CGAGCGGCCGCGCTTCAAGCCCTACTCTGCCC OR51I2 –forward: ATGCCCGCAACATCACTT OR51I2 –reverse: GCACAGGAGGCACAAATAGG TBP–forward: TATAATCCCAAGCGGTTTGC TBP–reverse: GCTGGAAAACCCAACTTCTG PLC—forward: AGGTTCAGGAGGATGTATGCC PLC–reverse: GCTCCTCGAAGTCTGCAGTT (DOCX) [file pone.0172491.s001.docx]

S1 Text: reverse transcriptase PCR (RT-PCR): The mixture contained 10 µl of GoTaq qPCR Master mix, 50 pmol Primers (fwd+rev) and cDNA (equivalent of 50 ng of total RNA). The PCR reaction contained the following steps: 3 min at 95°C followed by 40 cycles of 1 min at 59°C (OR51B4) or 60°C (PLC, TBP) and 1 min at 72°C and was performed in the Mastercycler® ep realplex (Eppendorf, Hamburg, Germany). TBP mRNA encoding the TATA-box binding protein was used for relative quantification by the ddCt method.

Primer:

*OR52D1 – forward: CCATGCTGGTGAGATTTCCT*

*OR52D1 – reverse: GGAGGCACCAGCACATAGAG*

*OR51B2 – forward: ACTGGATCTCCATCCCCTTC*

*OR51B2 – reverse: AGGGCTTTGGCTCTCTCTTC*

*OR51I1 – forward: ATGAGCTTGGATCGCTTTGT*

*OR51I1 – reverse: AAGCGGTGAATCATGGAGAC*

*OR51B5 – forward: GCAGGAGAGCAAAGAAGTCTC*

*OR51B5 – reverse: GGACAGGGGAAGGAGGTAAG*

*OR51B4 – forward*: CGAGAATTCAGCATGTGGTATAACAACAGTGC

*OR51B4 – reverse*: CGAGCGGCCGCGCTTCAAGCCCTACTCTGCCC

*OR51I2 – forward: ATGCCCGCAACATCACTT*

*OR51I2 – reverse: GCACAGGAGGCACAAATAGG*

*TBP – forward: TATAATCCCAAGCGGTTTGC*

*TBP – reverse: GCTGGAAAACCCAACTTCTG*

*PLC - forward: AGGTTCAGGAGGATGTATGCC*

*PLC – reverse: GCTCCTCGAAGTCTGCAGTT*
