# Supplementary material for: Activation of odorant receptor in colorectal cancer cells leads to inhibition of cell proliferation and apoptosis
Source: PLoS One. 2017 Mar 8;12(3):e0172491. doi: 10.1371/journal.pone.0172491 (PMC5342199; doi:10.1371/journal.pone.0172491)
Supplement: S2 Text — Geranonitrile, acetophenone, eucalyptol, thymol, anethole, menthol, camphor, muscone, citronellol, sweet orange oil, p-cymene, eugenol, geraniol, hedione, helional, lyral, d-carvone,l-carvone, citral a, benzyl acetate, sassafras oil, 15-pentadecanolide, methyl salicylate, linalool, phenylethyl alcohol, hexyl cinnamaldehyde (α), amyl cinnamaldehyde (α), iso-bornyl acetate, dihydro myrcene, benzyl salicylate, galaxolide, oil of turpentine, fixolide np, coumarin, styrlyl acetate, piperonal, jonone (β), ptbca 25 cis, traseolide, aldehyde c12, benzyl benzoate, cyclame aldehyde, dmbca, iso-nonyl acetate, benzophenone, bourgeonal, benzyl alcohol, otbca, cinnamyl alcohol, allyl heptanoate, oxyphenylon, cinnamaldehyde, agrunitril, brahmanol, citrathal, dimetol, epitone, iso-nonyl alcohol, phenylethyl acetate, phenirat, aldehyde c08, ethylfruitat, hexyl acetate, neobergamate, aldehyde c12, anisaldehyde, citrusal, cedryl acetate, ethylvanillin, evernyl, ligustral, dimedone, sandalore, vanillin, aldehyde 11–11, aldehyde 13–13, ambroxan, anthoxan, boisambrene forte, cyclohexyl salicylate, cyclovertal, floramat, herbavert, irotyl, jasmacyclat, melusat, peranat, romilat, sandelice, trivalon, troenan, verdoxan, propidyl, aldehyde c07, alcohol c08, amylbutyrate, prenylacetate, ethylamylketone, methylhexylketone, acedyl. (DOCX) [file pone.0172491.s002.docx]

**S2 Text: List of Henkel odorants in Henkel 100:**

Geranonitrile, acetophenone, eucalyptol, thymol, anethole, menthol, camphor, muscone, citronellol, sweet orange oil, p-cymene, eugenol, geraniol, hedione, helional, lyral, d-carvone,l-carvone, citral a, benzyl acetate, sassafras oil, 15-pentadecanolide, methyl salicylate, linalool, phenylethyl alcohol, hexyl cinnamaldehyde (α), amyl cinnamaldehyde (α), iso-bornyl acetate, dihydro myrcene, benzyl salicylate, galaxolide, oil of turpentine, fixolide np, coumarin, styrlyl acetate, piperonal, jonone (β), ptbca 25 cis, traseolide, aldehyde c12, benzyl benzoate, cyclame aldehyde, dmbca, iso-nonyl acetate, benzophenone, bourgeonal, benzyl alcohol, otbca, cinnamyl alcohol, allyl heptanoate, oxyphenylon, cinnamaldehyde, agrunitril, brahmanol, citrathal, dimetol, epitone, iso-nonyl alcohol, phenylethyl acetate, phenirat, aldehyde c08, ethylfruitat, hexyl acetate, neobergamate, aldehyde c12, anisaldehyde, citrusal, cedryl acetate, ethylvanillin, evernyl, ligustral, dimedone, sandalore, vanillin, aldehyde 11–11, aldehyde 13–13, ambroxan, anthoxan, boisambrene forte, cyclohexyl salicylate, cyclovertal, floramat, herbavert, irotyl, jasmacyclat, melusat, peranat, romilat, sandelice, trivalon, troenan, verdoxan, propidyl, aldehyde c07, alcohol c08, amylbutyrate, prenylacetate, ethylamylketone, methylhexylketone, acedyl.
