# Supplementary figures and images for: Activation of odorant receptor in colorectal cancer cells leads to inhibition of cell proliferation and apoptosis
Source: PLoS One. 2017 Mar 8;12(3):e0172491. doi: 10.1371/journal.pone.0172491 (PMC5342199; doi:10.1371/journal.pone.0172491)

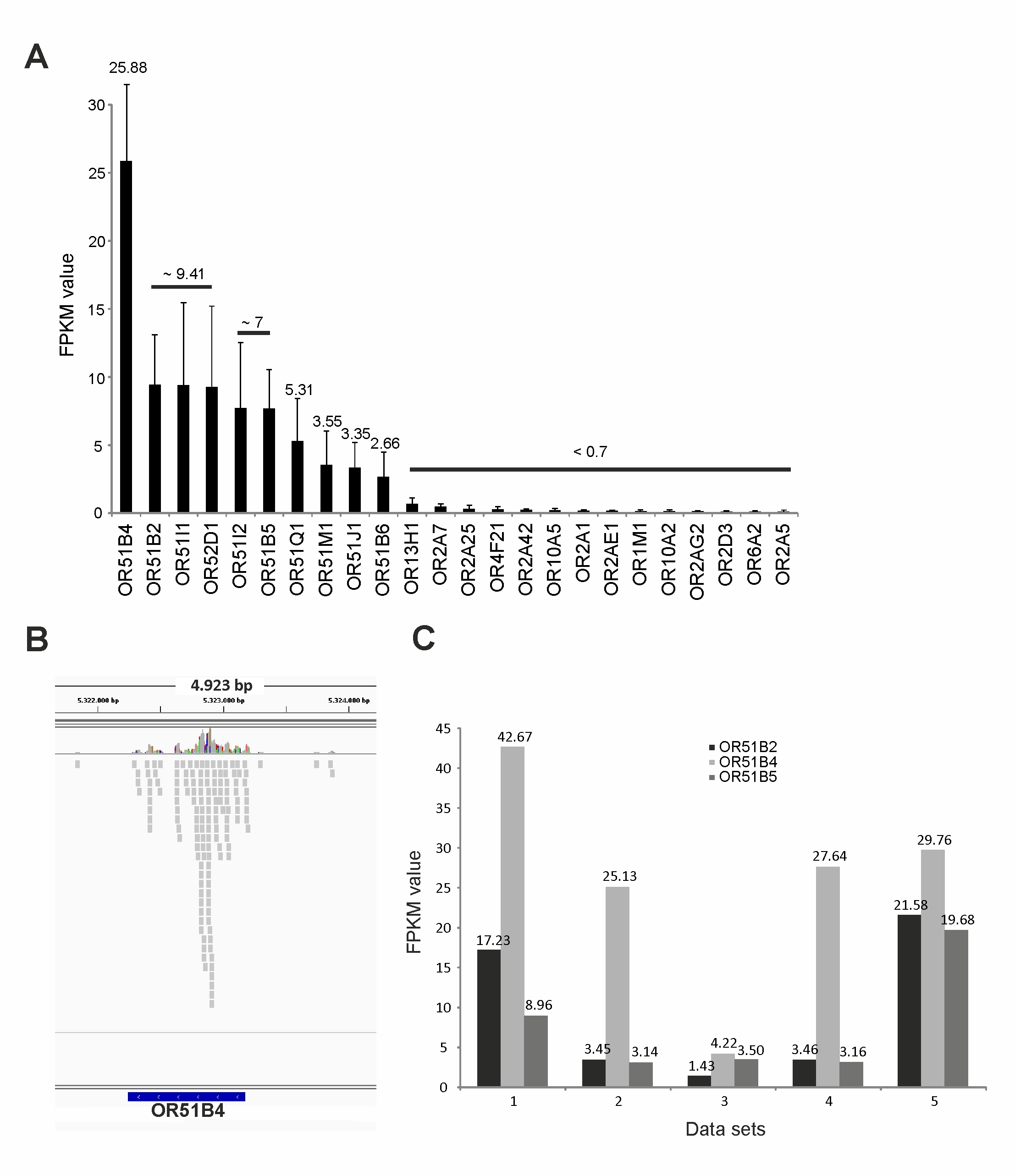

Supplement: S1 Fig — (A) Bar chart showing ranking of OR expression in HCT116 cells. (B) Read coverage of OR51B4 detected in HCT116 and visualized by the Integrative Genomic Viewer. (C) Average RNA-Seq transcriptome data for OR51B2, OR51B4 and OR51B5 from 5 different data sets. (TIF) [file pone.0172491.s003.tif]

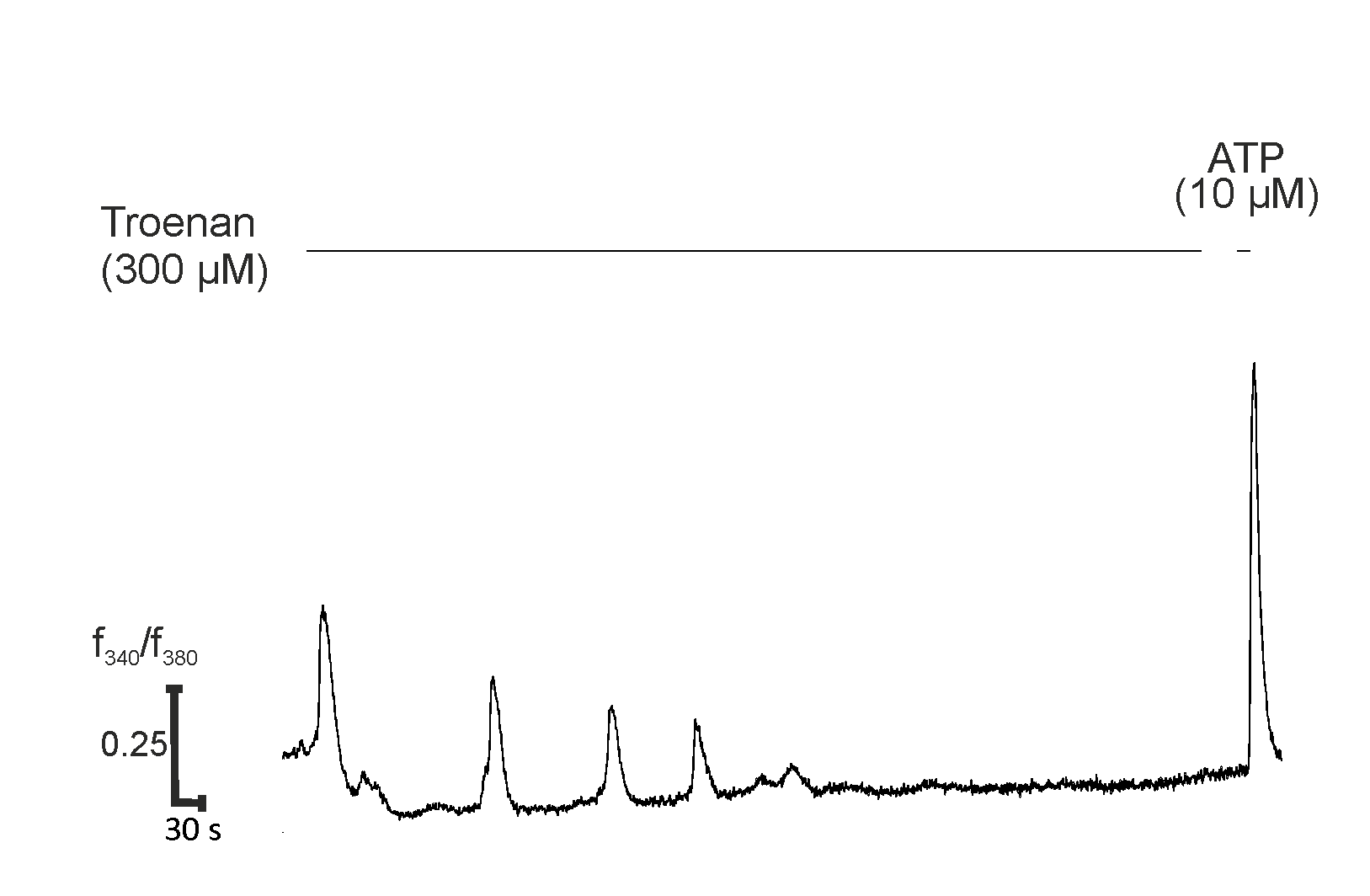

Supplement: S2 Fig — HCT116 cells exposed to Troenan (300 μM) for 20 minutes. ATP served as a control. (TIF) [file pone.0172491.s004.tif]

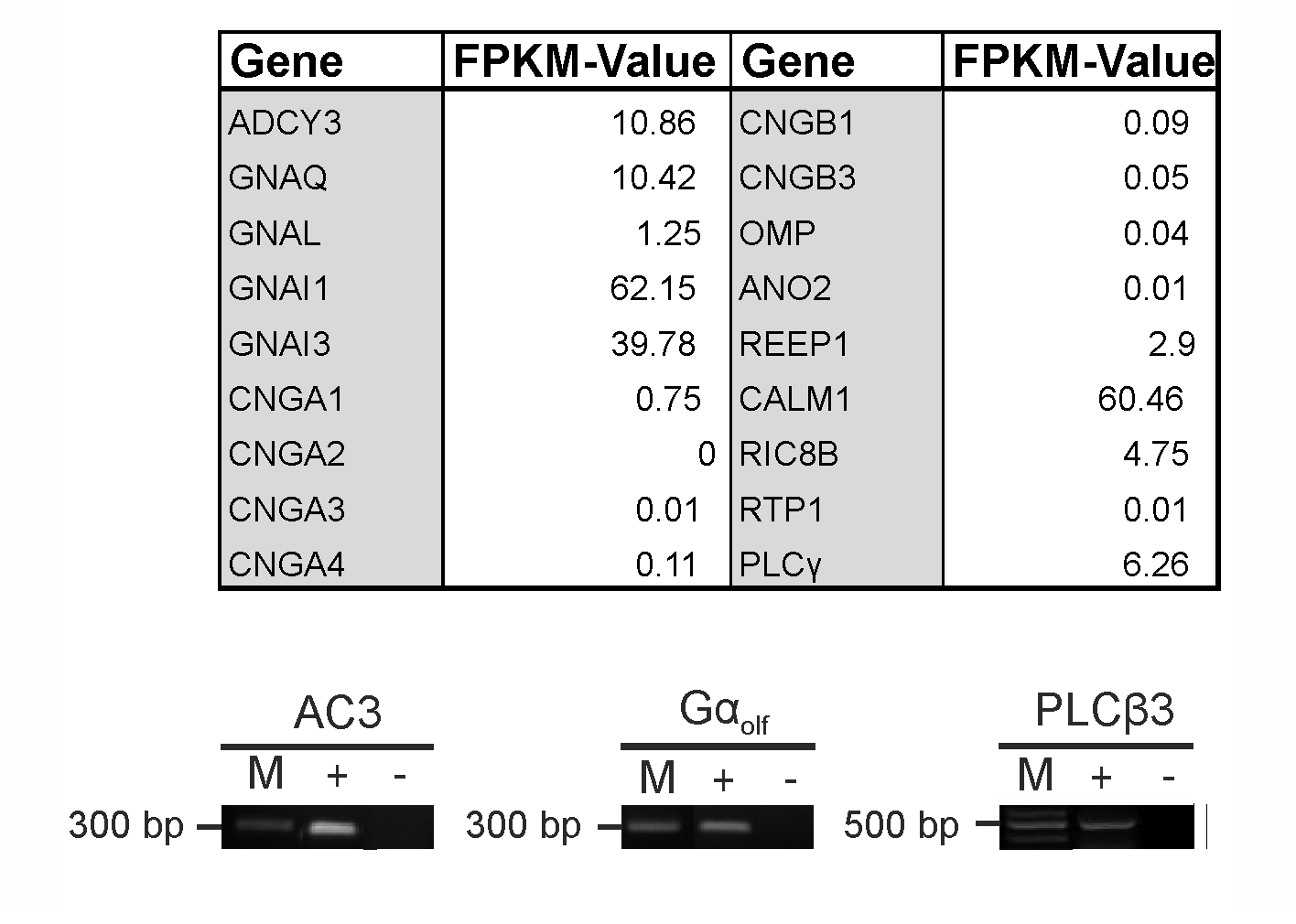

Supplement: S3 Fig — ADCY3: adenylyl cyclase 3, GNAQ: G-protein αq, GNAL: G-protein αolf, GNAI1/3: G-protein αi, CNGA1-4: CNG channel subunits (CNGA2, CNGA4, CNGA3, CNGA4, CNGB1 and CNGB3), OMP: olfactory marker protein, ANO2: calcium-activated chloride channel anoctamin 2, REEP1: receptor-enhancing proteins 1, CALM1: Calmodulin 1, RIC8B: nucleotide exchange factor, RTP1: receptor-transporting proteins. PLCγ: phospholipase C γ. PLCβ: phospholipase C β. (TIF) [file pone.0172491.s005.tif]
